# Detection and genetic characterization of atypical porcine pestivirus in wild boars in European Russia

**DOI:** 10.3389/fmicb.2026.1798555

**Published:** 2026-04-02

**Authors:** Afshona Anoyatbekova, Darya Zaugolnikova, Maria Dmitrieva, Oksana Kucheruk, Alexander Bulgakov, Anton Yuzhakov

**Affiliations:** Laboratory of Biochemistry and Molecular Biology, Federal State Budget Scientific Institution “Federal Scientific Center VIEV” (FSC VIEV), Moscow, Russia

**Keywords:** APPeV, genome, pestivirus, phylogenetic analysis, sequence, wild boar

## Abstract

Atypical porcine pestivirus (APPeV), currently known as *Pestivirus scrofae*, is common in domestic pigs of many countries in Europe, Asia, and America. In Russia, through a retrospective study, its circulation among domestic pigs has been confirmed since at least 2020. However, in the wild boar population, the presence of the virus remained unstudied. In this study a molecular survey was carried out in European Russia to investigate the virus circulation in the wild boars. In total, 445 tissue samples obtained from 236 wild boars hunted across seven regions of European Russia in the period of 2021–2025 were tested by qRT-PCR. The virus was found to be widespread among the population, with a total positive ratio of 9.7% (23/236). Specifically, APPeV was identified in wild boars across four regions (Moscow, Tver, Belgorod, and Tula), with detection rates ranging from 10.5 to 33.3%. It was established that the virus has been circulated in the wild boars since at least 2021. For phylogeny, a total of 13 partial sequences of the NS2-NS3 region were derived. Russian isolates exhibited high genetic variability and were distributed into three distinct clades. Two clades consisted solely of isolates identified in the present study. The nucleotide sequence identity between the Russian isolates varied from 86.1 to 99.1% and from 74.0 to 93.5% with strains from GenBank. To the best of our knowledge, this is the first report on APPeV circulation among wild boars in the territory of Russia.

## 1 Introduction

Atypical porcine pestivirus (APPeV), currently known as *Pestivirus scrofae*, is one of the 19 recognized species within the genus *Pestivirus* of the *Flaviviridae* family (Smith et al., 2017; ICTV, 2026). The virus was first discovered in North America in 2015 by next-generation sequencing in pig serum samples positive for porcine reproductive and respiratory virus (PRRSV) (Hause et al., 2015). Subsequent studies have established its widespread prevalence across many European and Asian countries as well as in North and South America (Beer et al., 2017; Lamp et al., 2017; Postel et al., 2017; Dessureault et al., 2018; Gatto et al., 2018; Kaufmann et al., 2019; Michelitsch et al., 2019; Xie et al., 2019; Dall Agnol et al., 2020; Hill, 2022; Kasahara-Kamiie et al., 2022). Initially, APPeV was not associated with the clinical disease development in infected pigs (Hause et al., 2015). Experimental infection of sows during gestation has demonstrated that the virus can induce congenital tremor (CT) type A-II in their offspring (Arruda et al., 2016). Soon thereafter, numerous studies associated APPeV with the appearance of the CT type A-II (de Groof et al., 2016; Postel et al., 2016; Dessureault et al., 2018; Sutton et al., 2019; Xie et al., 2019; Dall Agnol et al., 2020; Schumacher et al., 2021). Clinical manifestations were not apparent in the sows and weaned piglets, yet substantial viremia was present (Arruda et al., 2016; Postel et al., 2017; Kaufmann et al., 2019; Michelitsch et al., 2019; Sozzi et al., 2019; Pedersen et al., 2021; Song et al., 2023). The virus is capable of both horizontal and vertical transmission, yet its pathogenesis remains insufficiently studied (Arruda et al., 2016; Gatto et al., 2018; Dall Agnol et al., 2020).

APPeV is an enveloped, positive-sense, single-stranded (+ss) RNA virus with a genome of approximately 11.5 kb in size (Hause et al., 2015; ICTV, 2026). Its genome contains a single large open reading frame that encodes a polyprotein consisting of 3,635 amino acids processed into four structural proteins (C, Erns, E1, and E2) and eight non-structural proteins (Npro, P7, NS2, NS3, NS4A, NS4B, NS5A, and NS5B), which are flanked by 5′ and 3′ untranslated regions (UTRs) (Hause et al., 2015; Xie et al., 2019; Yuan and Wang, 2021).

It has been confirmed that there are significant genetic diversity and variability among APPeV strains in different countries (Postel et al., 2017; Dénes et al., 2018; Xie et al., 2019; Stenberg et al., 2022; Yuan et al., 2022), and homologous recombination was identified (Guo et al., 2020). Based on the complete genome sequences, the diversified APPeV strains have been divided into three clades, or genogroups (I, II, and III). In turn, seven subgenotypes have been identified for genotype I (1.1–1.7) (Yuan et al., 2017; Xie et al., 2019; Choe et al., 2020; Yuan and Wang, 2021). Yuan and Wang (2021) findings indicated that all strains of genotypes 2 and 3 originated exclusively from China. The genotype 1 comprised strains from China as well as strains from other countries. Consequently, the high degree of genetic diversity of APPeV strains found in domestic pigs, even within the same region, creates significant difficulties in determining their regional origin.

The wild boars (*Sus scrofa*) warrant a separate discussion. The data regarding APPeV infection in wild boars is scant. However, the virus has been detected in Germany, Italy, Serbia, Sweden, Spain, South Korea, and Japan (Cagatay et al., 2018; Colom-Cadena et al., 2018; Sozzi et al., 2019; Choe et al., 2020; Stenberg et al., 2022; Shiokawa et al., 2025). The transmission routes from wild boars to pigs or vice versa have not been studied yet. Most APPeV-positive wild boars remained asymptomatic, with no pathological lesions observed in their organs during post-mortem examination (Colom-Cadena et al., 2018).

It is known that wild boars play a crucial role in the transmission of many infectious diseases, thus posing a threat to the pig industry (Meng et al., 2009; Reiner et al., 2009; Jezdimirović et al., 2024). Accordingly, a deep insight into the pathogens’ circulation in wild boars is required to develop preventive and control strategies, to safeguard public health, and to strengthen biosecurity in pig farms. Given that wild boars are ubiquitous throughout Russia’s various geographical areas, ongoing surveillance remains essential, especially for effective control of African swine fever virus (ASFV) (Pershin et al., 2019; Mazloum et al., 2022; Anastasia et al., 2025). Besides ASFV, the circulation of porcine parvoviruses, porcine circoviruses, classical swine fever virus (CSFV), and Aujeszky’s disease virus has been reported in the Russian wild boar population (Shcherbakov et al., 2007; Krasnikov et al., 2024; Komina et al., 2025). Notably, APPeV presence in Russian wild boars has not been investigated. Recently, our studies indicated that APPeV has been prevalent in Russian pig populations since at least 2020, with an overall prevalence rate of 8.8% (Anoyatbekova and Yuzhakov, 2024).

Hence, this study aimed to provide molecular evidence of APPeV in wild boars habituated in the territory of European Russia.

## 2 Materials and methods

### 2.1 Investigation areas and sampling

During the 2021–2025 hunting seasons, a total of 236 free-ranging wild boars hunted in the territory of seven regions of European Russia, including Moscow (*n* = 124), Belgorod (*n* = 25), Tver (*n* = 41), Tula (*n* = 6), Lipetsk (*n* = 28), Ryazan (*n* = 3), and Krasnodar Krai (*n* = 9), were investigated. From these animals, 445 tissue samples [lungs (*n* = 92), spleen (*n* = 231), and lymph nodes (*n* = 122)] were obtained (Supplementary Table S1). The samples were collected year-round by hunters with special permission from the hunting grounds of the region. Data from the sampled wild boars, including gender, weight, and age, were estimated visually by the hunters, and the shot date and location were recorded. Ages were estimated based on the eruption of teeth but not for all the sampled animals. In total, age was determined for 67 young wild boars (<1 year old) and 42 adults (>1 year old). Gender was indicated for 82 wild boars. There were no data available on clinical manifestations of any infections or postmortem changes in organs. All collected material was previously tested for the presence of ASFV and CSFV and confirmed to be negative in the regional laboratories and subsequently was sent to the laboratory of Biochemistry and Molecular Biology of the Federal Scientific Center (FSC) All-Russian Institute of Experimental Veterinary (VIEV). Samples were transported on ice in Styrofoam containers and subsequently stored at −70 °C until use.

### 2.2 Samples processing, RNA extraction and qRT-PCR

Tissue samples in an amount of 0.3–0.5 g were ground thoroughly in 2.5 mL of sterile PBS in a 15 mL centrifuge tube (Nest, Wuxi, China) and centrifuged at 3000 × g for 20 min at 8 °C. The supernatant was collected and used for RNA extraction using “RiboPrep” (FBIS Central Research Institute of Epidemiology of Rospotrebnadzor, Moscow, Russia) following the manufacturer’s protocol. All tissue samples were tested individually. The extracted RNA was either used immediately or stored at −70 °C until further use.

For reverse transcription quantitative real-time polymerase chain reaction (qRT-PCR), primers and probes of the 5’UTR region of APPeV described by Kaufmann et al. (2019) were used. qRT-PCR was performed as described previously (Anoyatbekova and Yuzhakov, 2024). To determine concomitant infection with PCV2-3 and PPV1, we utilized data from prior investigations conducted in our laboratory under the framework of RSC grant No. 23-76-10055, parts of which have been previously published (Krasnikov et al., 2024; Komina et al., 2025).

### 2.3 Reverse transcription, PCR and sequencing

Reverse transcription and PCR procedures were performed independently. The cDNA was synthesized using random hexamer primers and the Superscript IV kit (Thermo Fisher Scientific, Invitrogen) as per the manufacturer’s protocol. Primers for the NS2-NS3 region designed by Postel et al. (2016) were used for sequencing. We carried out nested PCR using BioMaster HS-Taq Color (2x) (BiolabMix, Moscow, Russia) with primers APPV_4186-fw/APPV_5169-rev (1st step) and APPV_4273-fw/APPV_5169-rev (2nd step) with the following temperature profiles: 95 °C for 5 min, followed by 38 cycles of denaturation at 95 °C for 20 s, annealing at 58 °C for 20 s, elongation at 72 °C for 40 s, and final elongation at 72 °C for 10 min. The PCR product was analyzed in 1% agarose gel electrophoresis containing Tris-acetate buffer solution (pH = 8.0) and ethidium bromide (0.5 mg/mL).

The PCR products of APPeV-positive samples with 896 bp in size were purified via the Monarch PCR & DNA Cleanup Kit (New England Biolabs, Ipswich, MA, USA) following the manufacturer’s protocol. The purified DNA was sequenced in both directions using the Big Dye 3.1 Terminator Cycle Sequencing Kit (Thermo Fisher Scientific, Carlsbad, CA, USA) following the manufacturer’s instructions and carried out on the ABI PRISM 3130 Genetic Analyzer (Thermo Fisher Scientific, Carlsbad, CA, USA) sequencing device.

### 2.4 Phylogenetic analysis

The obtained sequences were processed using SeqMan Lasergene 11.1.0 (DNASTAR, Madison, WI, USA). The phylogeny was performed based on the analysis of the obtained partial sequences of the NS2-NS3 region in MEGA 7.0. Sequences were aligned by the MUSCLE algorithm. The phylogenetic tree was constructed using the maximum likelihood (ML) method based on the general time reversible (GTR), (G + I) model. The topology evaluation was performed by 1,000 bootstrap replications, and pairwise genetic distances were calculated following the Tamura 3-parameter model. Phylogenetic analysis was conducted using 32 sequences retrieved from GenBank and 13 sequences newly derived in this study.

### 2.5 Cell cultures and virus isolation

Continuous cell cultures of the porcine embryo kidney cell line (SPEV) and immortalized pig spleen cells (SIPS) from the “Specialized collection of continuous, somatic cell cultures of domestic and wild animals at the Federal Scientific Centre VIEV” (FSC VIEV Cell Collection, Moscow, Russia) were used for the APPeV isolation. Primary porcine testicular cells (PPTC) were derived from piglets 3 weeks old following the previously described method (Anoyatbekova et al., 2025). The SPEV cell culture was maintained in 199 Medium (PanEco, Moscow, Russia) with 10% heat-inactivated bovine serum (Biosera, Cholet, France), 10 U/mL penicillin, and 10 μg/mL streptomycin (PanEco, Moscow, Russia). The SIPS were grown in Dulbecco’s modified Eagle’s medium with 4,5 g/L glucose (PanEco, Moscow, Russia) supplemented with 10% fetal bovine serum (FBS) (IntlKang, Beijing, China), 2 mM L-glutamine, and 10 U/mL of penicillin and 10 μg/mL of streptomycin (PanEco, Moscow, Russia). The cell lines were cultured in 25 cm^2^ tissue culture flasks for 3 days post-seeding at 37 °C. The 0.25% trypsin–EDTA (PanEco, Moscow, Russia) was used for the cell’s dissociation from the flasks. Prior to infection, continuous and primary cell cultures were confirmed to be free of BVDV, CSFV, and *Mycoplasma spp*. in qPCR using commercial kits (Vetbiochem and BiolabMix, Moscow, Russia).

For APPeV isolation in cell culture, we used samples with the lowest Cq (17.4–20.0) identified in the diagnostic PCR and free of contaminant infections (PCV2-3 and PPV1). Tissue samples after homogenization were transferred to a 50 mL sterile centrifuge tube (Nest, Wuxi, China) and centrifuged at 3000 × g for 20 min at 4 °C. From the supernatant a 10% suspension was prepared and kept in Eagle medium containing 10 units/mL penicillin and 10 μg/mL streptomycin (PanEco, Moscow, Russia) for 2 h at 4 °C. Then it was filtered through the 0.20 μm syringe filter and used as stock material for virus isolation. Virus isolation was conducted following the method described earlier (Anoyatbekova and Yuzhakov, 2024). For mock infection Eagle medium was used.

### 2.6 Statistical analysis

The APPeV detection rate was calculated as the ratio of positive samples to the total number of samples analyzed. Categorical variables (age groups, seasons, gender, and tissue types) were compared using the *χ*^2^ test or Fisher’s exact test, where appropriate. Statistical significance was defined as *p* < 0.05. All statistical analyses were performed using GraphPad Prism v8 and Past 4.17 software.

## 3 Results

### 3.1 Geographical distribution of APPeV in the investigated areas

**Figure 1:**
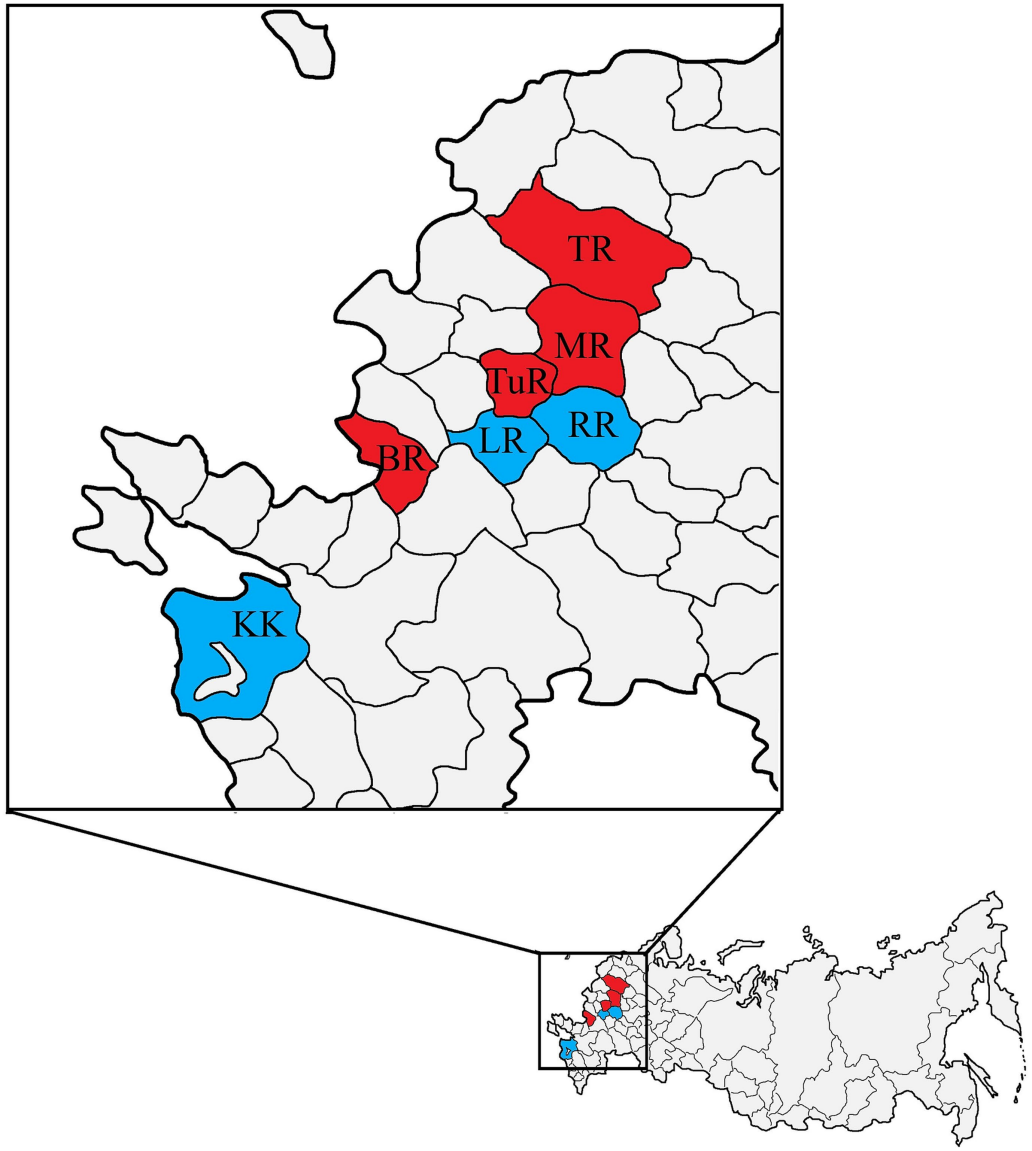
Geographical distribution of APPeV in the investigated regions. In the figure the European Russia is presented. In total, 236 wild boars were hunted through seven regions: TR–Tver Region; MR–Moscow Region; TuR–Tula Region, LR–Lipetsk Region; RR–Ryazan Region, LR–Lipetsk Region; KK–Krasnodar Krai. The regions with APPeV-positive wild boars are marked in red, while those with negative ones are marked in blue.

**Figure 2:**
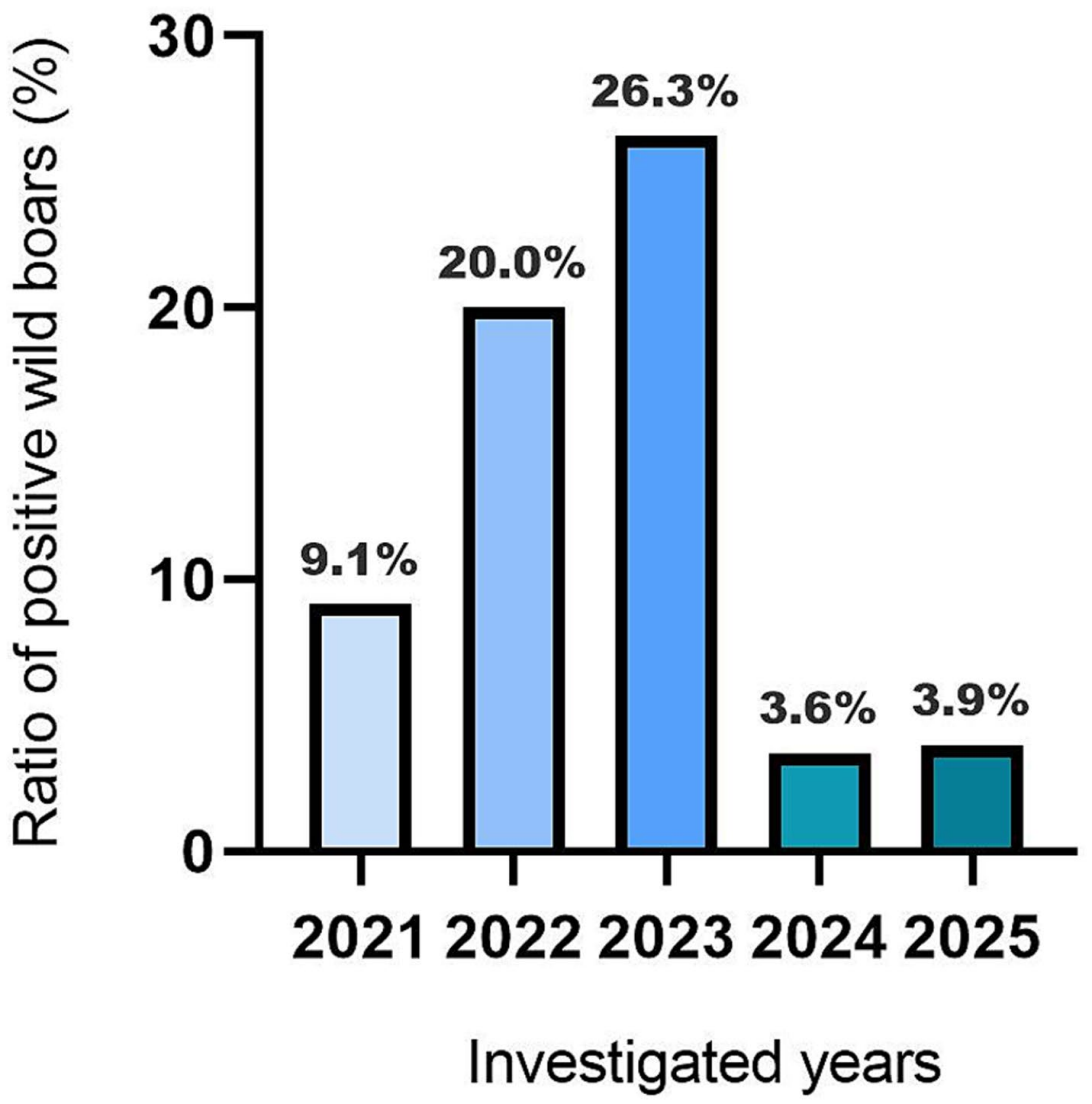
APPeV detection rates by years in the Moscow region.

Out of 236 wild boars sampled from seven regions of European Russia, 9.7% (23/236) tested positive by qRT-PCR in four regions (57.1%) (Table 1). Geographically, all regions (Moscow, Tver, Belgorod, and Tula) where APPeV-positive wild boars were found lie in the western part of European Russia (Figure 1). Virus detection rates varied across regions, spanning from 10.5% in the Moscow region to 33.3% in the Tula region. The virus was not detected in wild boars from the Ryazan and Lipetsk regions and the Krasnodar Krai. Statistically significant differences in the virus detection rates among regions were not observed (*p* > 0.05) and cannot be reliably assessed due to the small sample sizes in some regions. Moreover, the temporal distribution of APPeV across regions could not be established, as wild boar samples were collected at only one or two time points (Supplementary Table S1). The Moscow region was the only one wherein wild boars samples (Supplementary Table S2) were obtained during the entire study period (2021–2025). Following results, the virus detection rate varied significantly over the five-year period with statistically significant differences (p < 0.05). From 2021 to 2023, a growing pattern in virus detection rate was evident (Figure 2). The peak of virus spread in the area was observed in 2023, reaching 26.3% (5/19). Nevertheless, a sharp decline to 3.6–3.9% was noted for 2024–2025, respectively.

**Table 1:** APPeV detection rates in the investigated regions.

Investigated areas	Wild boars			Samples		
	Total	APPeV-positive	% Positive	Total	APPeV-positive	% Positive
Moscow	124	13	10.5	232	21	9.1
Tver	41	5	12.2	87	6	6.9
Belgorod	25	3	12.0	50	4	8.0
Ryzan	3	0	0	8	0	0
Tula	6	2	33.3	17	4	23.5
Lipetsk	28	0	0	42	0	0
Krasnodar Krai	9	0	0	9	0	0
Total/7	236	23	9.7	445	35	7.9

### 3.2 APPeV distribution by tissue species, age groups, gender and season

Among 445 samples collected from 236 wild boars, 35 samples tested positive for APPeV, representing a 7.9% detection rate (Table 1). The total threshold cycle of the virus in the lungs ranged from 20.21 to 34.81 Cq, in the spleen from 29.5 to 33.14, and in the lymph nodes from 17.04 to 31.27, respectively. A complete set of organs (lungs, lymph nodes, and spleen) was obtained from 66 wild boars, and APPeV was detected in 20 of them.

The age of wild boars was established for a mere 109 individuals across seven regions. All wild boars from birth to <1 year old were classified as juveniles, while those older (>1 year) were classified as adults. The viral genome was identified in the samples of both tested age groups, with a different detection rate ranging from 9.5 to 13.4% (Table 2). Sex was indicated for only 82 wild boars (Table 2). To determine the seasonal pattern, tested wild boars were divided by season in accordance with the shooting day. APPeV in the hunting grounds was detected year-round. No statistically significant differences in the level of virus detection rates among age groups, gender, season, and tissue species were observed (*p* > 0.05).

**Table 2:** APPeV-positivity by characterized groups.

Characteristics	Total in groups	Positive (%)
Seasons		
Winter (December to February)	64	7 (10.9%)
Spring (March to May)	57	2 (3.5%)
Summer (June to August)	18	2 (11.1%)
Autumn (October to November)	97	15 (15.5%)
Age categories		
Juveniles (<1 year)	67	9 (13.4%)
Adults (>1 year)	42	4 (9.5%)
Gender		
Male	22	4 (18.2%)
Female	60	8 (13.3%)
Tissue species		
Lungs	66	6 (9.1%)
Spleen	66	7 (10.6%)
Lymph nodes	66	7 (10.6%)

### 3.3 Phylogenetic analysis of APPeV isolates

**Figure 3:**
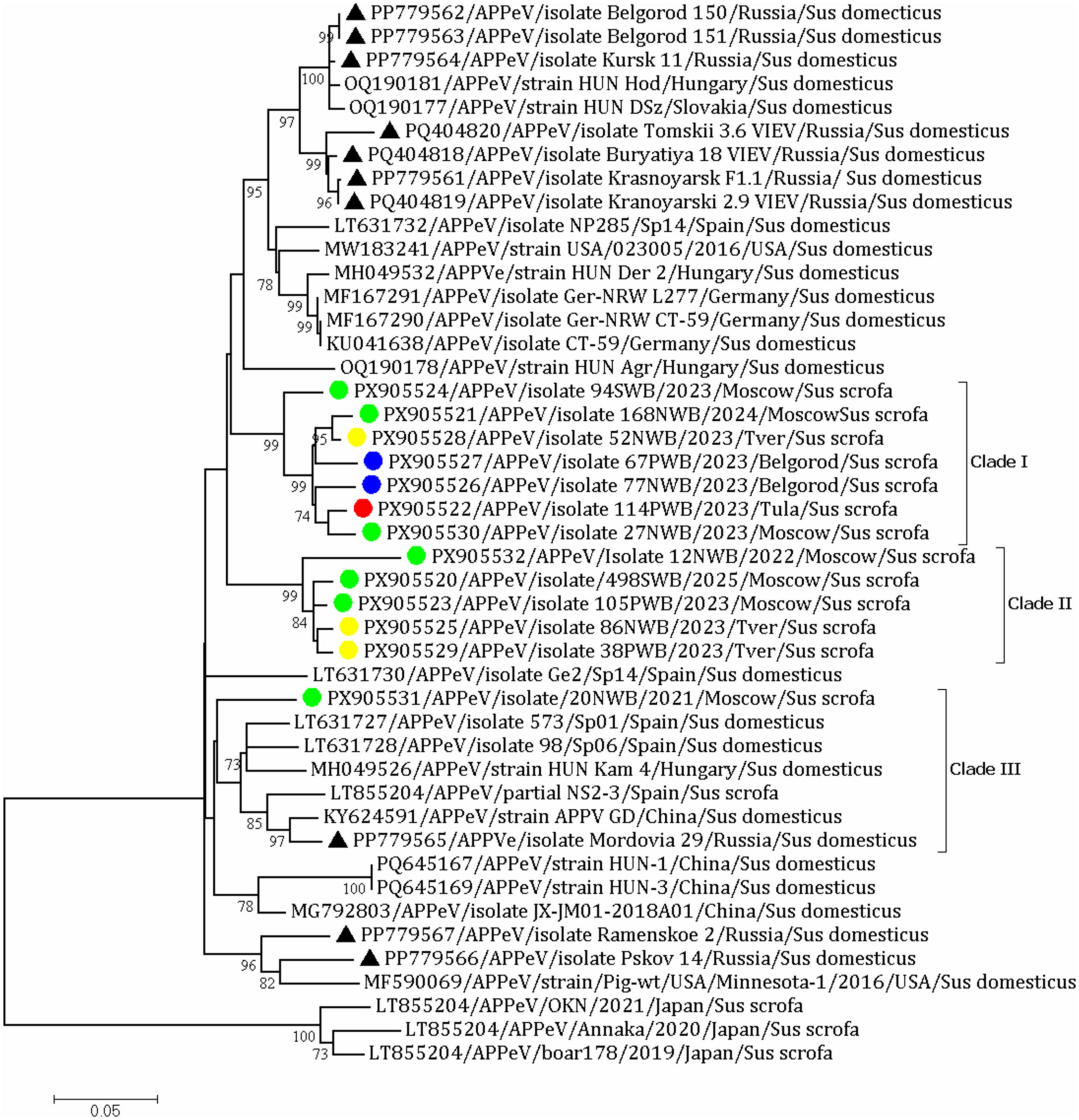
The phylogenetic tree of a partial NS2-NS3 gene of the APPV strains from GenBank and Russian isolates. The dendrogram was constructed by the ML method and the GTR model (G + I). Bootstrap support values (≥70) are provided. The scale bar indicates 0.05 expected changes per site per branch. The APPV sequences obtained in this study are indicated by circle. Different colors represent different regions; green circles for isolates from Moscow region; yellow circle for isolates from Tver region; blue circle for isolates from Belgorod region; red circle for isolate from Tula region. The APPeV isolates derived from domestic pigs are designated by ▲.

In total, 13 partial sequences of NS2-NS3 were derived in this study: seven from the Moscow region, two from Belgorod, three from Tver, and one from the Tula region. Phylogenetic analysis revealed three clades (Clades I-III) encompassing all Russian isolates in this study. Twelve of these isolates clustered in two distinct clades (Clades I-II) and highly varied from known sequences (Figure 3). Previously identified isolates from Russian domestic pigs did not group with those in this study. The overall nucleotide sequence identity between the Russian isolates from wild boars varied from 86.1 to 99.1% and from 74.0 to 93.5% with APPeV strains from GenBank.

The first clade included three isolates from the Moscow region (PX905521, PX905524, PX905530), one from the Tver region (PX905528), one from the Tula region (PX905522), and two from the Belgorod region (PX905526 and PX905527). The nucleotide sequence identity among them ranged from 89.6 to 96.7%. Three isolates originated from the Moscow region (PX905520, PX905523, and PX905532), and two from the Tver region (PX905525 and PX905529) formed Clade II with 93.3–94.5% nucleotide sequence identities. Clade III comprised the APPeV/20NWB/2021/Moscow isolate (PX905531), identified in wild boar in the Moscow region. Additionally, this clade included isolates originating from Spain (LT631727, LT631728, and LT855204), Hungary (MH049526), China (KY624591), and a Russian isolate from Mordovia (PP779565) earlier identified in domestic pigs.

Isolates from the Moscow region exhibited the highest genetic diversity, being distributed across all three identified clades with nucleotide sequence identities ranging from 86.8 to 99.1%. Notably, these sequences showed low identity (87.3–91.9%) to a previously reported domestic pig isolate from the same area (PP779567). The sequences of Tver isolates (PX905525, PX905529) shared 91.1–91.5% identity, while those from the Belgorod (PX905526, PX905527) and Tula (PX905522) regions were more closely related, sharing 97.0–97.3% nucleotide similarity.

### 3.4 Co-infection of APPeV with economically significant pathogens

All wild boars (*n* = 236) were tested for the presence of PCV2-3, PPV1, and PRRSV. There were not any positive cases with PRRSV. Among APPeV-positive wild boars (Table 3), a substantial 78.2% (18/23) demonstrated co-infection with other pathogens. Specifically, dual infections involving APPeV/PPV1 and APPeV/PCV3 each occurred in 8.7%, while APPeV/PCV2 was considerably more frequent at 34.8% of cases. Mixed infections, with APPeV/PPV1/PCV2 and APPeV/PCV2/PCV3, composed 4.3 to 17.4%, respectively. A notable 21.7% of the analyzed cases presented with APPeV as a mono-infection.

**Table 3:** Co-infections of APPeV with economically significant porcine viruses.

Investigated viruses	The number of APPeV-positive wild boars with mono- and co-infection	The percentage of APPeV-positive wild boars with mono- and co-infection
APPeV	5	21.7%
APPeV+PPV1	2	8.7%
APPeV+PCV2	8	34.8%
APPeV+PCV3	2	8.7%
APPeV+PPV1 + PCV2	1	4.3%
APPeV+PPV1 + PCV3	0	0%
APPeV+PCV2 + PCV3	4	17.4%
APPeV+PPV1 + PCV2 + PCV3	1	4.3%

### 3.5 APPeV isolation in cell culture

To isolate APPeV, continuous (SPEV and SIPS) and primary cell cultures (PPTCs) were used. Accordingly, for virus isolation, the samples with the lowest Cq and free of concurrent infections were selected. Virus isolation was attempted from lymph nodes of wild boars hunted in the Moscow and Belgorod regions. During daily microscopy we did not observe any morphological changes in the infected and mock-infected cells. Virus cultivation was continued for 6–7 days post-infection, and its replication was assessed using qRT-PCR. Three blind passages were conducted in all cell cultures. The results demonstrated that all passages conducted in SIPS and SPEV are APPeV-negative. In PPTCs we detected the viral genome throughout three passages; however, at a low virus concentration and with a subsequent increase of Cq (Table 4). All mock-infected cell cultures were APPeV-negative.

**Table 4:** APPeV replication in infected PPTCs cell culture.

Isolate	Source of isolation	Initial Cq	1st passage	2nd passage	3rd passage
APPeV/27NWB/2023/Moscow (PX905530)	Lymph nodes	20.48	27.38	35.04	36.62
APPeV/77NWB/2023/Belgorod (PX905526)	Lymph nodes	17.14	28.72	35.65	36.01

The Cq during APPeV/27NWB/2023/Moscow (PX905530) cultivation ranged from 27.38 to 36.62 and APPeV/77NWB/2023/Belgorod (PX905526) cultivation from 28.72 to 36.01, respectively. As the Cq increased following three passages, the 4th virus passage was not conducted.

## 4 Discussion

Wild boar (*Sus scrofa*) are the mammals with the highest reproductive rate and exceptional environmental adaptability globally. Due to the common species designation (*Sus scrofa*), the wild boars and domestic pigs share similar susceptibility to the same pathogens and are capable of interspecies transmission of the disease (Meng et al., 2009; Malmsten et al., 2017; Johann et al., 2020). This poses a risk to the pig farming industry by spreading infections among the animals (Hayama et al., 2020; Anastasia et al., 2025; Zakharova et al., 2025). Pig farming is one of the main agricultural sectors in Russia; thus, regular genetic surveillance of viruses is critical to minimize potential economic losses. Recently, we have confirmed the widespread circulation of APPeV in domestic pig herds in Russia (Anoyatbekova and Yuzhakov, 2024). Nevertheless, its presence and prevalence within wild boar populations have not been studied until now. In light of the aforementioned points, the present study was designed to investigate the circulation pattern of APPeV within the wild boar populations in Russia.

European Russia has been designated the focus of our investigation, as significant large-scale pig farming industries are concentrated within its borders. Moreover, the abundance of food and forests in this geographical area brings wild boars into contact with agricultural lands, thereby increasing their population density. It has been determined that in the European territory of Russia, the wild boar population density is estimated to be 1.006 animals/km^2^ (Zakharova et al., 2025). Our study yielded 445 tissue samples from 236 wild boars, hunted in seven regions of European Russia over a period of 2021 and 2025. Following qRT-PCR, the APPeV genome was detected in the wild boar population in four regions (Moscow, Tver, Belgorod, and Tula) with an overall detection rate of 9.7% (23/236), which is higher than that of domestic pigs (8.8%; 232/2630) in Russia (Anoyatbekova and Yuzhakov, 2024). The virus detection rate in Russian wild boars is a little less than in Sweden, which amounted to 12% (73/595) (Stenberg et al., 2022). APPeV prevalence was notably high in the German wild boar population, with 19.0% (86/456) (Cagatay et al., 2018). In stark contrast, in Spain (0.22%, 1/437), Italy (0.69%, 3/430), South Korea (0.78%, 18/2297), and Japan (0.6%, 2/333), significantly lower rates of viral genome detection were reported in wild boars (Colom-Cadena et al., 2018; Sozzi et al., 2019; Choe et al., 2020; Shiokawa et al., 2025). Besides detecting the viral genome in wild boars’ serum in Germany and Sweden, high seropositivity rates, standing at 52% (238/456) and 72% (433/595), were observed, respectively (Cagatay et al., 2018; Stenberg et al., 2022). The proportion of positive detection rates exhibits considerable variability from one country to another. These disparities are recognized to be influenced by a multitude of factors, including population density, climate, fencing, agricultural and forestry methods as well as hunting practices, the number of wild boars studied, and sample species (Hayama et al., 2020; Stenberg et al., 2022; Zakharova et al., 2025). In the present study, the number of wild boars sampled exhibited considerable regional variation. The absence of statistical significance may be attributable to limitations in sample size within certain investigated areas. Therefore, to substantiate the hypotheses proposed, further comprehensive investigations are warranted, wherein sampling effort should be proportionally aligned with the estimated wild boar population density in each specific region.

For phylogenetic analysis, we attempted to obtain sequences from all positive samples. However, due to the low virus load, we were unable to sequence all of them. Since the NS2-NS3 region is considered to be highly conserved for *Pestiviruses* (Postel et al., 2016; Beer et al., 2017; Dénes et al., 2018), we used this particular segment for sequencing. Overall, we derived 13 partial sequences, with at least one sample from each region. Sequences from GenBank databases from other countries, as well as previously characterized Russian isolates of domestic pigs, were included in the phylogenetic analysis. Our findings indicated that isolates from wild boars are highly variable, forming independent clades (Clades I-II) separate from other groups. Genetic diversity among isolates was obvious, not only when comparing those from different countries but also within a specific geographical area. This was exemplified by findings from the Tver and Moscow regions, where distinct viral variants were concurrently circulating within a single geographic area.

This heterogeneity is typical for APPeV (Yuan et al., 2017; Yan et al., 2019; Guo et al., 2020). In China three genotypes (1–3) and seven subgenotypes within genotype 1 (1.1–1.7) in domestic pigs were proposed (Yuan et al., 2017; Yan et al., 2019; Yuan and Wang, 2021). High genetic diversities of APPeV in domestic pigs were also confirmed in Germany, Spain, and Italy (Postel et al., 2017; Cagatay et al., 2018). Apart from the single isolate APPeV/20NWB/2021/Moscow (PX905531), the remaining isolates detected in this study did not cluster with any of the previously identified Russian pig isolates (Anoyatbekova and Yuzhakov, 2024). These findings are consistent with the Cagatay et al. (2018) studies, reporting that isolates identified in German wild boars formed their own separate phylogenetic clade and were genetically distinct from isolates collected from pigs in Germany (Cagatay et al., 2018). Conversely, in the studies of Colom-Cadena et al. (2018) in Spain, Choe et al. (2020) in Korea, Stenberg et al. (2022) in Sweden, and Shiokawa et al. (2025) in Japan, APPeV was detected in wild boars grouped in one clade together with isolates of domestic pigs, and thus the possibility of interspecies transmission was suggested. Because sequencing targeted different genomic regions, an extensive comparative analysis of our isolates with those obtained from wild boars in other countries could not be undertaken. Thus, establishing an epidemiological or genetic link between them is not possible at this stage. As well as in the case of the APPeV/20NWB/2021/Moscow isolate (PX905531), which had high nucleotide identity with the Mordovian isolate (PP779565) earlier identified in pigs despite the fact that these regions are geographically located in European Russia. It can be hypothesized, by analogy with other viruses, that the finding of multiple genetic clades of a single virus in a single region could reflect either varied introduction methods or the virus’s endemic presence, where evolving lines outcompete and replace each other (Creanga et al., 2013; Blome et al., 2017; Zakharova et al., 2025).

Of all the regions studied, the Moscow region was the only one wherein all samples from wild boars were obtained throughout the entire study period (2021–2025). The temporal analysis provided insight into the fact that in the wild boars hunted in European Russia, APPeV has circulated since at least 2021, although it was detected in pigs in the Krasnoyarsk Krai in 2020 (Anoyatbekova and Yuzhakov, 2024). This underscores the need for comprehensive virus monitoring in the wild boar population, covering not only the European Russia but also all other regions of their habitat. Between 2021 and 2023, a substantial growth from 9.1 to 26.3% (*p* < 0.05), followed by a sharp decline in 2024, with a subsequent slight rise occurring in 2025 in the virus-positive rate, was noticed. The observed significant reduction in virus detection rates among the wild boars from 2024 to 2025 lacks a definitive explanation and might depend on various factors. It can be hypothesized that this decline occurred due to the depopulation of wild boars as a consequence of the epizootic measures implemented to control ASFV (Pershin et al., 2019; Anastasia et al., 2025; Zakharova et al., 2025). Alternatively, it can be assumed that the development of herd immunity led to a decrease in active APPeV transmission, thereby improving animal immune response to the virus. However, to clarify this, it is necessary to investigate the wild boars’ serum samples for the presence of antibodies.

Hunting in Russia is carried out in strict compliance with federal law, which grants regions autonomy in this matter (Anastasia et al., 2025). When analyzing the accompanying documentation, it was determined that in the regions of European Russia, wild boar hunting is carried out throughout the year. To determine the seasonal pattern, we divided all wild boars into groups according to the month of shooting. The findings established that APPeV in the investigated regions circulates year-round in the wild boar population. These patterns are in line with the data provided by Stenberg et al. (2022). Our findings indicate that APPeV does not exhibit any correlation with seasonality, age, or sex of wild boars. Although age and sex were not determined for all wild boars in our study, the results demonstrated that the virus can infect both adults and juveniles of both sexes, which is consistent with studies in pigs (Arruda et al., 2016; Kaufmann et al., 2019; Dall Agnol et al., 2020; Pedersen et al., 2021; Song et al., 2023). A Swedish investigation suggests that young wild boars are particularly susceptible to APPeV infection, attributed to their high levels of social interaction within and between groups. However, no clinical signs of infection have been reported in wild boars’ offspring (Stenberg et al., 2022). Despite analyzing samples derived from multiple tissue types, the present study does not include data pertaining to the clinical course or pathological findings associated with infection. In total, 445 samples from 236 wild boars were analyzed, but the entire organ set (lungs, spleen, and lymph nodes) was available from only 66 boars. From the remaining animals, either two or one sample of each type was obtained. There was no statistically significant variation in the frequency of virus detection in tissue samples (*p* > 0.05), indicating that the virus is present in all organs, but the target organ remains unknown. Given the preceding discussion, a thorough examination of the clinical and pathological presentations is essential.

It’s worth noting that the frequent co-infection of APPeV with other pathogens may complicate the investigation of its pathological features. It is known that APPeV was first identified in pigs co-infected with the PRRS virus (Hause et al., 2015). Subsequent investigation in pigs observed its co-infection with PCV2, PCV3, astrovirus, etc. (Lamp et al., 2017; Dall Agnol et al., 2020). Sozzi et al. (2019) reported a co-infection of APPeV and PCV3 in wild boars. This finding is consistent with our data, given that we detected PCV3 in 8.7% of APPeV-positive wild boars. Furthermore, we revealed dual infection with PCV2 (34.8%), and PPV1 (8.7%). Additionally, a mixed infections involving APPeV/PPV1/PCV2 and APPeV/PCV2/PCV3 were detected. It has to be mentioned that in 21.7% of wild boars, APPeV mono-infection was observed. The obtained results hold scientific importance and warrant additional investigation since it is unknown whether co-infection with other viruses impacts the course of APPeV infection.

Upon isolation of APPeV variable isolates from Russian European wild boars in cell culture, further *in vivo* investigations of the virus will be enabled. The successful isolation of the virus necessitates the presence of viral samples exhibiting a high viral load and being free from any concurrent viral infections. Recent studies conducted by Shiokawa et al. (2025) in Japan have successfully demonstrated the isolation of viruses from wild boars. Our investigation also included attempts to isolate the virus in cell cultures. However, we noted a decline in viral concentration across passages within primary PPTS cell cultures; thus, the subsequent passage was not carried out. Furthermore, continuous cell cultures failed to support viral replication, as confirmed by qRT-PCR results. This finding diverges from our previous study, which reported virus replication in continuous cells (SPEV and SIPS) (Anoyatbekova and Yuzhakov, 2024). It should be noted that the viral material was derived from blood serum, and parallel attempts to isolate the virus from pathological specimens were likewise unsuccessful. Based on the literature data and our previous study, it can be assumed that APPeV-positive sera have a higher success rate for virus isolation compared to pathological material (Beer et al., 2017; Cagatay et al., 2019; Anoyatbekova and Yuzhakov, 2024). Accordingly, our upcoming investigations will continue to pursue the isolation of the virus from diverse sample types through cell culture systems.

## 5 Conclusion

To our knowledge, this is the first large-scale study to investigate the circulation of APPeV among wild boars in Russia and confirm its wide distribution in the European part. This underscores the need for comprehensive virus monitoring in wild boar populations across all regions of Russia. The results of phylogenetic analysis revealed high variability among Russian wild boar isolates and require enhanced study and in-depth phylogenetic analysis to trace the source of each specific APPeV variant and prevent their further spreading. Further *in vitro* investigations are essential to identify the optimal conditions for virus cultivation and to derive a stable cultural strain.

## Data Availability

The datasets presented in this study can be found in online repositories. The names of the repository/repositories and accession number(s) can be found at: https://www.ncbi.nlm.nih.gov/genbank/, PX905920-PX905932.
