# Supplementary material for: Detection and genetic characterization of atypical porcine pestivirus in wild boars in European Russia
Source: Front Microbiol. 2026 Apr 2;17:1798555. doi: 10.3389/fmicb.2026.1798555 (PMC13083189; doi:10.3389/fmicb.2026.1798555)
Supplement: Supplementary file 1 [file Table_1.docx]

Supplementary Table 1 (ST1). **APPeV detection rates in investigated areas**

| **Investigated areas /regions** | **Total  number  of samples from wild boars** | **2021** | **2022** | **2023** | **2024** | **2025** | **Total  number  of wild  boars** |  |
| --- | --- | --- | --- | --- | --- | --- | --- | --- |
|  |  |  |  |  |  |  |  |  |
|  | **positive/total/%** | | | | | | |  |
| **Moscow** | **21/232/9.1** | **2/11/18.2** | **3/15/20.0** | **5/19/26.3** | **1/28/3.6** | **2/51/3.9** | **13/124/10.5** |  |
| **Tver** | **6/87/6.9** | 0/0/0 | 0/0/0 | **5/35/14.3** | **0/6/0** | 0/0/0 | **5/41/12.2** |  |
| **Belgorod** | **4/50/8.0** | 0/0/0 | 0/0/0 | **4/25/16** | 0/0/0 | 0/0/0 | **3/25/12.0** |  |
| **Ryzan** | **0/8/0** | 0/0/0 | 0/0/0 | **0/1/0** | **0/2/0** | 0/0/0 | **0/3/0** |  |
| **Tula** | **4/17/23.5** | 0/0/0 | 0/0/0 | **2/4/50.0** | 0/0/0 | **0/2/0** | **2/6/33.3** |  |
| **Lipetsk** | **0/42/0** | 0/0/0 | 0/0/0 | **0/28/0** | 0/0/0 | 0/0/0 | **0/28/0** |  |
| **Krasnodar Krai** | **0/9/0** | 0/0/0 | 0/0/0 | **0/9/0** | 0/0/0 | 0/0/0 | **0/9/0** |  |
| **Positive 4/total 7 /57.1%** | **35/445/7.9** | **2/11/18.2** | **3/15/20.0** | **16/121/13.2** | **1/36/2.7** | **2/53/3.8** | **23/236/9.7** |  |
