# Supplementary material for: Detection and genetic characterization of atypical porcine pestivirus in wild boars in European Russia
Source: Front Microbiol. 2026 Apr 2;17:1798555. doi: 10.3389/fmicb.2026.1798555 (PMC13083189; doi:10.3389/fmicb.2026.1798555)
Supplement: Supplementary file 2 [file Table_2.docx]

| **Years** | **Total number of wild boars** | **Positive** | **%** | **95 % confidence interval (%)** |
| --- | --- | --- | --- | --- |
| 2021 | 11 | 2 | 18.2 | 5.14-48.0 |
| 2022 | 15 | 3 | 20.0 | 7.05-45.19 |
| 2023 | 19 | 5 | 26.3 | 11.81-49.36 |
| 2024 | 28 | 1 | 3.6 | 0.63-17.72 |
| 2025 | 51 | 2 | 3.9 | 1.08-13.01 |
| Total | 124 | 13 | 10.5 | 6.24-17.10 |

Supplementary Table 2 (ST2). **APPeV detection rates in the Moscow region by years**
